# A High Protein Model Alters the Endometrial Transcriptome of Mares

**DOI:** 10.3390/genes10080576

**Published:** 2019-07-30

**Authors:** Yatta L. Boakari, Hossam El-Sheikh Ali, Pouya Dini, Shavahn Loux, Claudia B. Fernandes, Kirsten Scoggin, Alejandro Esteller-Vico, Laurie Lawrence, Barry Ball

**Affiliations:** 1 Maxwell H. Gluck Equine Research Center, Department of Veterinary Science, University of Kentucky, Lexington, KY 40546, USA; 2 Theriogenology Department, University of Mansoura, Mansoura 35516, Egypt; 3 Faculty of Veterinary Medicine, Ghent University, B-9820 Merelbeke, Belgium; 4 Department of Animal Reproduction, University of São Paulo, São Paulo 05508-270, Brazil; 5 Department of Biomedical and Diagnostic Sciences, University of Tennessee, Knoxville, TN 37996, USA; 6 Department of Animal Science, University of Kentucky, Lexington, KY 40546, USA

**Keywords:** high protein diets, high blood urea nitrogen concentrations, urea, uterus, horse, early embryonic loss, fetal loss

## Abstract

High blood urea nitrogen (BUN) decreases fertility of several mammals; however, the mechanisms have not been investigated in mares. We developed an experimental model to elevate BUN, with urea and control treatments (7 mares/treatment), in a crossover design. Urea-treatment consisted of a loading dose of urea (0.03 g/kg of body weight (BW)) and urea injections over 6 h (0.03 g/kg of BW/h). Control mares received the same volume of saline solution. Blood samples were collected to measure BUN. Uterine and vaginal pH were evaluated after the last intravenous infusion, then endometrial biopsies were collected for RNA-sequencing with a HiSeq 4000. Cuffdiff (2.2.1) was used to identify the differentially expressed genes (DEG) between urea and control groups (false discovery rate-adjusted *p*-value < 0.1). There was a significant increase in BUN and a decrease of uterine pH in the urea group compared to the control group. A total of 193 genes were DEG between the urea and control groups, with five genes identified as upstream regulators (*ETV4, EGF, EHF, IRS2*, and *SGK1*). The DEG were predicted to be related to cell pH, ion homeostasis, changes in epithelial tissue, and solute carriers. Changes in gene expression reveal alterations in endometrial function that could be associated with adverse effects on fertility of mares.

## 1. Introduction

Early embryonic and fetal losses affect reproductive efficiency and productivity in farm animals [1]. Nutritional imbalance and/or disorders are some of the factors that could lead to these losses. Several studies have shown that a high protein diet results in an increase in blood urea nitrogen (BUN), associated with lower fertility in cows and ewes [2,3,4,5,6]. 

Cows receiving a high protein diet exhibited an increase in systemic BUN and a decrease in uterine luminal pH at day 7 of the estrous cycle (diestrus) [7,8]. This alteration in the uterine environment is critical, as by this time the embryo would have reached the uterus and the endometrium would be undergoing remodeling to prepare for the early conceptus [9]. When urea, a metabolite of protein digestion, was given intravenously or orally to cows and ewes during the luteal phase, conditions mimicked those of a high protein diet, including an acute elevation of BUN, an increase in uterine urea, and a decrease in uterine pH [2,5,7,8,10,11,12,13]. Additionally, the in vitro effects of high urea concentrations on bovine endometrial explant from diestrus resulted in altered endometrial gene expression related to cell growth, proliferation, and differentiation [14]. It has been suggested that there might be deleterious effects of an altered endometrial tissue and uterine environment on early embryonic development due to a high BUN in animals [14,15]. For instance, a lower media pH resulted in detrimental consequences to bovine embryos cultured in vitro [16]. Overall, these studies showed that a high protein diet caused an increase in systemic BUN which was associated with an increase in intrauterine urea, and a decrease in uterine pH. 

Even though high protein diets might have deleterious effects on reproduction in cows and ewes, it is part of normal nutrient management practices to feed an excessive amount of crude protein (CP) to horses [17,18]. This excessive amount of CP might result in BUN concentrations above the normal range for horses that may result in negative consequences to the uterine environment. However, no studies to date have addressed the effects of a high protein diet on fertility in the mare.

It is important to study the effects of a high BUN in the endometrium of mares, as broodmares might be routinely receiving a high CP diet. To the best of our knowledge, there are no published studies regarding the influence of elevated BUN on the endometrial transcriptome of mares. Therefore, we hypothesized that an acute intravenous infusion of urea would elevate BUN with a concomitant decrease in uterine pH resulting in transcriptomic changes in the endometrium of mares. The objectives of the present study were to (1) develop a model to elevate BUN, (2) correlate changes in BUN to changes in uterine pH, and (3) evaluate how changes in circulating BUN affected the endometrial transcriptome of mares. 

## 2. Material and Methods

All animal procedures were completed in accordance to the Institutional Animal Care and Use Committee of the University of Kentucky (Protocol #2011-0876). Clinically healthy mares of mixed breeds, ranging from 5 to 15 years of age were used in this study. All mares underwent a reproductive examination and transrectal ultrasonography for reproductive tract evaluation (vulva, cervix, uterus, and ovaries) before the experiment. The researchers only used mares with no detectable abnormalities of the reproductive system. 

Mares received a treatment or control infusion (n = 7 mares/group) in a crossover design. The intervening estrous cycle was skipped and served as a washout cycle. Mares received 2500 IU of human chorionic gonadotropin (hCG) (Chorulon; Intervet, Millsboro, DE, USA) intravenously when they had a follicle of at least 35 mm in diameter and pronounced uterine edema as determined by transrectal ultrasonography (ExaGo; ECM Co., Angouleme, France). Animals were scanned daily by ultrasound for ovulation detection (Day 0 = ovulation) and infusions were initiated at day 7 of diestrus (D7). On the day of infusion, both jugular veins were catheterized using a 14 G x 2” gauge indwelling catheter (NIPRO Medical Corporation, Miami, FL, USA). One jugular catheter was used for blood collection, and the opposite jugular catheter was used for infusion of urea (treatment) or saline (control). Treatment consisted of a loading dose of 0.03 g/kg of body weight (BW) of urea (Sigma-Aldrich Company, St. Louis, MO, USA) diluted in 100 mL of saline solution (Hospira, Inc, Lake Forest, IL, USA) (15 g of urea for a 500 kg horse) to achieve a rapid increase in urea concentrations, and control mares received 100 mL of saline solution. Subsequently, mares received a bolus injection of urea (0.03 g/kg of BW/hr) diluted in 15 mL of saline solution every 30 min over 6 h (90 g of urea over 6 h for a 500 kg horse). The control group received the same amount of saline solution as the urea group, 100 mL of saline solution as a loading dose and 15 mL of saline solution every 30 min over 6 h. 

### 2.1. Blood Urea Nitrogen Concentration

Blood samples were collected hourly in 10 mL vacutainer tubes with sodium heparin (BD Vacutainer, Franklin Lakes, NJ, USA). Blood samples were promptly centrifuged at 1500× *g* for 10 min at 4 °C, and plasma was stored at −20 °C. BUN was measured with a colorimetric spectrophotometric assay following an adapted protocol previously described [19]. All reagents were purchased from Sigma-Aldrich. The standard curve ranged from 5.6 mg/dL to 56.0 mg/dL. Urea was diluted (8 M after constitution with 16 mL high purity water) to 5.6 mg/dL and 56.0 mg/dL to be used as low and high controls. The reaction consisted of analyzing urea by enzymatic hydrolysis to ammonia at room temperature. The reaction was done in microcentrifuge tubes (2 mL) with 10 μL of each plasma sample in duplicate and 125 μL urease buffer was added, and the samples were incubated for 20 min. The urease enzyme hydrolyzes urea to produce carbon dioxide and ammonia (CH_4_N_2_O + Urease buffer → CO_2_ + 2NH_3_). Then, 250 μL of phenol nitroprusside solution, 250 μL of alkaline hypochlorite solution (0.2%), and 1000 μL of distilled water were added (NH_3_ + phenol nitroprusside + alkaline hypochlorite + H_2_O → Indophenol blue) [20]. After a 25-min incubation, a 200 μL aliquot was transferred to a 96-well plate and read in an Epoch microplate spectrophotometer plate reader (Biotek, San Francisco, CA, USA) at 570 nm. The intra- and inter-assay coefficients of variation for BUN concentrations were 0.5% and 9.8%, respectively. The lower limit of detection of the assay was 0.11 mg/dL.

### 2.2. Uterine and Vaginal pH

Uterine and vaginal pH were measured immediately after the last intravenous infusion with urea or saline solution. The mares were restrained in palpation stocks, and their tails were wrapped and tied. Feces were removed from the rectum manually. The perineal region was washed three times with povidone-iodine scrub, rinsed with clean water, and dried with clean paper towels. An adapted epoxy pH probe attached to a portable pH meter (Thermo Fisher Scientific, Waltham, MA, USA) was used for pH measurements. Immediately before pH measures, the probe was calibrated with calibration solution buffers at pH 4, 7, and 10 (Thermo Fisher Scientific). The pH probe was introduced into the vagina with the tip protected by a sterile gloved hand and passed through the cervix. The pH probe was advanced into the uterus until it reached the uterine body and was held in place by the examiner. The examiner introduced the other hand into the rectum to increase the contact surface between the uterine wall and the pH device. When the uterine pH measurement was completed, the pH probe was removed from the uterus and placed into the vagina, in contact with the vaginal mucosa next to the cervix. The pH meter probe was maintained in the same position until two stable reads were completed. This was done two times, in the uterus and vagina, and the mean of the readings was calculated.

### 2.3. Endometrial Biopsies 

After uterine pH measurements, the perineal region was washed again three times with povidone-iodine scrub, rinsed with clean water and dried with clean paper towels. A Jackson uterine biopsy forceps designed for horses (60-cm length, 4 mm × 28 mm cut-off area, Jorgensen Laboratories, Inc., Loveland, CO, USA) was guarded in a sterile gloved hand and passed through the cervix into the uterus. A uterine biopsy was collected from the base of the uterine horn. The sample was removed from the instrument with a sterile needle (NIPRO Medical Corporation), and preserved in RNAlater (Thermo Fisher Scientific), kept at 4 °C overnight and then kept at −80 °C until RNA isolation [21]. Mares received dinoprost tromethamine (5 mg, im; Lutalyse; Pfizer, New York, NY, USA) to help with uterine clearance after intrauterine procedures. 

### 2.4. RNA Extraction

Total cellular RNA was extracted from endometrial samples using TRIzol Reagent (Thermo Fisher Scientific) following the manufacturer’s recommendations. After extraction, RNA concentration and quality were analyzed using a NanoDrop DP-1000 spectrophotometer (Agilent Technologies, Palo Alto, CA, USA) and a Bioanalyzer^®^ (Agilent, Santa Clara, CA, USA). All samples had a 260/280 ratio > 2.0 and RNA integrity number (RIN) > 8 (8.95 ± 0.4, mean ± SEM). A total of 1 μg of RNA was treated with DNase I (Ambion Inc., Austin, TX, USA) for 30 min at 37 °C to remove genomic DNA according to manufacturer’s instructions. The extracted RNA was kept at −20 °C until further analyses.

### 2.5. mRNA Library Preparation and Next Generation Sequencing 

The extracted RNA (1 μg), as described above, was sent to the University of Illinois at Urbana-Champaign for library preparation and RNA Sequencing. Paired-end reads with 150 nucleotides in length were produced. The RNAseq libraries were prepared with Illumina’s TruSeq Stranded mRNAseq Sample Prep kit (Illumina, San Diego, CA, USA). Read 1 aligns to the antisense strand and Read 2 aligns to the sense strand. The libraries were quantitated by qPCR and sequenced on one lane for 101 cycles from each end of the fragments on a HiSeq 4000 using a HiSeq 4000 sequencing kit version 1. The lane produced a total of 700 million reads. Fastq files were generated and demultiplexed with the bcl2fastq v2.17.1.14 Conversion Software (Illumina). 

### 2.6. Next Generation Sequencing Data Analysis

The data discussed in this publication are accessible through GEO Series accession number GSE131810 (https://www.ncbi.nlm.nih.gov/geo/query/acc.cgi?acc=GSE131810). The Fastq files were evaluated for read quality using FastQC 0.11.4 [22]. Subsequently, Trim Galore 0.4.1 [23] was used for adapter and read quality trimming (Phred score threshold of 30). Reads were mapped to the *Equus caballus* reference genome (EquCab 3.0) using the software STAR 2.5.3a [24], then annotated with the equine reference annotation from NCBI using Cufflinks 2.2.1 [25]. Fragments per kilobase per million (FPKM) were used to determine the expression level of genes. Lastly, we used Cuffdiff 2.2.1 [25] to calculate differentially expressed genes (DEG) between samples from the control and urea groups. Significance level was set at false discovery rate-adjusted *p*-value of the test statistic <0.1 using a Benjamini-Hochberg correction. 

### 2.7. Functional Annotation and Pathway Analysis

The Database for Annotation, Visualization, and Integrated Discovery Bioinformatics Resources (DAVID, version 6.8, https://david.ncifcrf.gov/home.jsp) was used to annotate DEG in relation to biological process, molecular function, and cellular component [26]. A functional classification was performed based on the Official Gene Symbol of *Equus caballus* genes in DAVID. GOplot (http://wencke.github.io/) was used to illustrate the results. DAVID was used to describe the functions of DEG based on public genomic resources through gene-set enrichment.

A core analysis of the DEG was conducted using ingenuity pathway analysis (IPA, QIAGEN Inc., https://www.qiagenbioinformatics.com/products/ingenuitypathway-analysis) as this commercial software package uses networks based on cause and effect relationships reported in previous studies. A diseases and biological functions analysis was done to show networks of biological interest. Additionally, an upstream regulator analysis was conducted to identify molecules that are upstream of the genes in this study that affect and help to explain the changes in expression observed. The *p*-value of overlap was calculated using a Fisher’s exact test (*P* < 0.01) to identify regulators that might explain the changes in expression of other genes [27,28]. 

The R-based weighted correlation network analysis (WGCNA) package was used to evaluate the correlation patterns among the genes in this RNA-sequencing experiment [29,30]. The FPKM data was transformed to log2(x + 1) prior to the analysis to normalize the data. WGCNA was used to generate clusters of highly interconnected genes identified by different colors, called modules. A power of 10 was chosen because it was the lowest possible power term that topology fitted a scale free network. Additionally, genes that were highly connected in the modules were identified as hub genes. 

### 2.8. Quantitative Real-Time PCR

Expression levels of a subset of DEG determined by RNA sequencing between the control and urea groups were confirmed with RT-qPCR. The extracted RNA was reverse transcribed using a high-capacity cDNA reverse transcription kit and random hexamers (Thermo Fisher Scientific). The cDNA was kept frozen at −20 °C until quantitative real-time PCR (RT-qPCR) was done. Primers for the selected transcripts were designed using the Primer-BLAST (National Center for Biotechnology Information, NCBI) function (Supplementary Table S1). The RT-qPCR was done using PowerUp™ SYBR™ Green Master Mix (Thermo Fisher Scientific) with the program: 95 °C for 10 min, 40 cycles of 95 °C for 15 s and 60 °C for 1 min, and 55–95 °C for dissociation cycling conditions. Each reaction was done in duplicate.

The RT-qPCR efficiency was determined using LinRegPCR (version 2012.0) to ensure that it was between 1.8 and 2.2 [31]. Mean threshold cycles were used to show changes in the mRNA expression and then normalized to the housekeeping genes Beta-2-Microglobulin (*B2M*) and Eukaryotic Translation Elongation Factor 1 Alpha 1 (*EEF1A1*) to calculate delta CT values (ΔCT) [32]. The two housekeeping genes were chosen with GeNORM [33] as the most stably expressed genes in the endometrial samples.

### 2.9. Statistical Analyses

The BUN, uterine, and vaginal pH and RT-qPCR were tested for normality with a Shapiro–Wilk test. The BUN concentration was not normally distributed and a normal quantile transformation was done. A fit least squares model using hour, treatment, and interaction between hour and treatment with mare as a random effect was used, followed by a Student’s t-test. The uterine and vaginal pH data had a normal distribution and were analyzed with a one-tailed paired t-test. Pearson’s correlation coefficients were done between the BUN concentration at the end of the treatment (H6) and the uterine pH and also between the uterine and vaginal pH.

A Pearson’s correlation coefficient was used to determine the correlation between the −ΔCT (negative delta CT) from RT-qPCR results and the FPKM from RNA-sequencing results. Data was reported as mean ± SEM. Significance was set at *P* ≤ 0.05 and trend at 0.1 > *P* > 0.05. JMP Pro (version 14; SAS Institute, Cary, NC, USA) was used for all statistical tests.

## 3. Results

### 3.1. Blood Urea Nitrogen Concentrations

**Figure 1:**
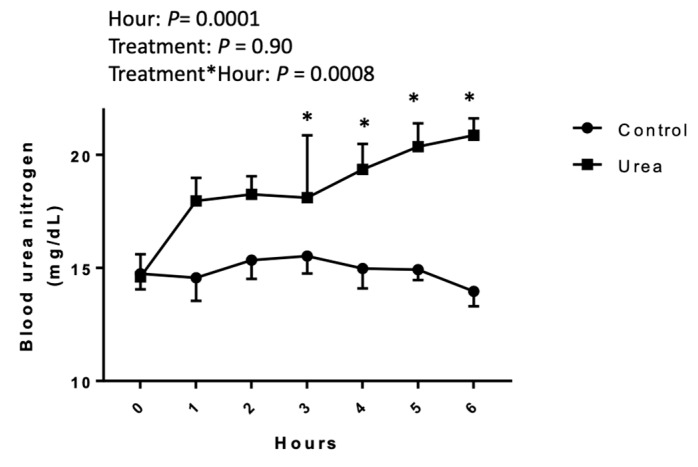
Blood urea nitrogen (mg/dL) analyzed in diestrus mares receiving an intravenous control or urea treatment over 6 h in a crossover design. A fit least squares model was used, followed by a Student’s t-test. Results are presented as mean and SEM. The main effect of hour, treatment, and interaction are shown. * *P* ≤ 0.05.

There was an effect of time of sampling (*P* < 0.0001) and of the interaction between time of sampling and treatment (*P* = 0.0008). There was no treatment effect (*P* = 0.90). Immediately before the start of the treatment (H0), the urea and control groups had BUN concentrations of 14.26 ± 0.69 and 14.12 ± 0.99 mg/dL in the control and treated mares, respectively, with no statistical difference (*P* > 0.05). The BUN at H6 was 14.33 ± 0.66 and 20.36 ± 0.75 mg/dL in the control and treated mares, respectively, showing a statistical difference (*P* < 0.05) (Figure 1). 

### 3.2. Uterine pH

**Figure 2:**
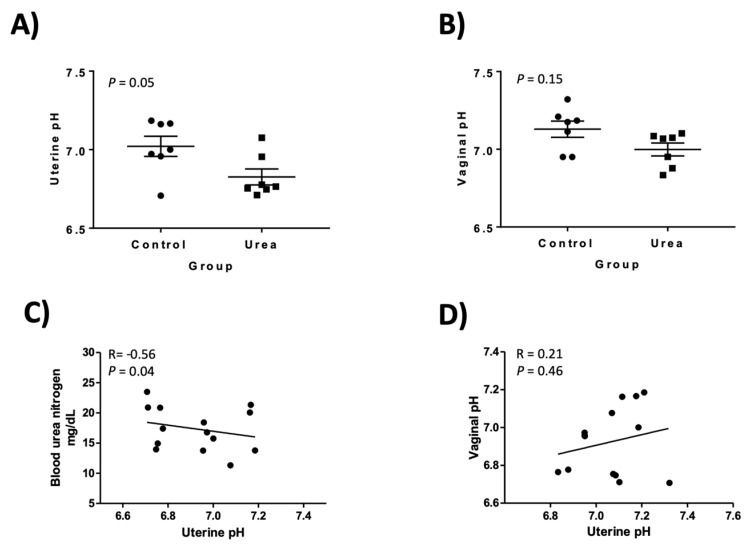
Uterine pH (**A**) and vaginal pH (**B**) analyzed in diestrus mares receiving an intravenous control or urea treatment over 6 h done in a crossover design. Horizontal line represents mean ± SEM. (**C**) Correlation between blood urea nitrogen (BUN, mg/dL) concentrations at hour 6 and uterine pH. (**D**) Correlation between uterine and vaginal pH at hour 6.

Based upon a one-tailed paired t-test there was a decrease in uterine pH in the urea group (*P* = 0.05). Uterine pH was 7.02 ± 0.06 and 6.83 ± 0.05 pH in the control and treated group at H6, respectively (Figure 2). There was a negative correlation (R = −0.56, *P* = 0.04) between the BUN at H6 and uterine pH (Figure 2).

### 3.3. Vaginal pH

Based upon a one-tailed paired t-test the vaginal pH was not different between the treated and control group (*P* = 0.15). Vaginal pH was 7.13 ± 0.05 and 6.99 ± 0.11 in the control and treated group at H6, respectively (Figure 2). The correlation between uterine and vaginal pH was not significant (R = 0.21, *P* = 0.46, Figure 2).

### 3.4. RNA Sequencing

The RNA-sequencing analysis performed on 14 endometrial samples resulted in 18,950 genes. The average of input reads per sample was 28,045,588 ± 1,768,799; the input read length for paired end reads was 150 and 95.5% of uniquely mapped reads were obtained for the samples sequenced. 

### 3.5. Differentially Expressed Genes

A total of 193 genes were differentially expressed between the urea and control groups. A total of 29 were upregulated and 162 were downregulated in the urea group in comparison to the control group. Additionally, 2 differentially expressed genes (DEG), aldo-keto reductase family 1 member C23 (*AKR1C23*) and alpha-fetoprotein (*AFP*) were of particular interest, as they had no expression in the urea group, only in the control group. 

Genes that were uncharacterized in the NCBI database had their nucleotide sequence (FASTA format) identified in the NCBI database (http://www.ncbi.nlm.nih.gov/), then the basic local alignment search tool (BLAST, http://www.ncbi.nlm.nih.gov/BLAST) [34] was used to identify their orthologs in other species (*Canis lupus dingo*, *Equus asinus*, *Equus przewalskii*, *Homo sapiens*) (all DEG are shown in the Supplementary Table S2). 

### 3.6. Functional Analyses

**Figure 3:**
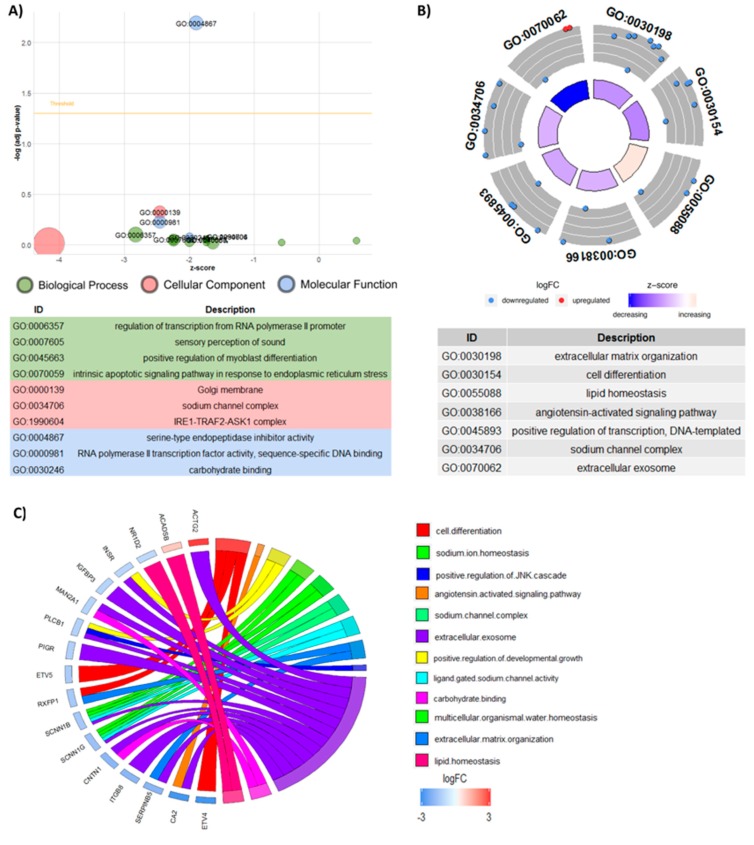
Gene ontology of differentially expressed genes analyzed by DAVID and the GOplot package. (**A**) GOBubble plot of differentially expressed genes analyzed by DAVID. The y-axis shows the −log10 (adjusted *P*-value) and the x-axis shows the z-score. The area of the circles is proportional to the number of genes related to the term. A threshold for the labeling is set based on the negative logarithm of the adjusted *p*-value. The Biological Process, Cellular Component and Molecular Function are represented by green, pink and blue, respectively. The identification (ID) and description of each term is given. (**B**) GOCircle plot showing the number of genes in each GO term. The inside rings are a bar plot, with the height representing the significance of the term (-log10 adjusted *P*-value). Different colors of the inside rings represent the z-score, with blue showing a decrease and red an increase. The outside rings show the expression levels (logFC) for each gene in the GO term. Each dot in the outside rings represents one gene from the GO term, red dots show upregulated genes and blue dots show downregulated genes. (**C**) GOChord plot show genes linked by ribbons to their respective GO terms and the logFC of each gene. The colored squares next to each gene represent the logFC, with red showing positive logFC, blue showing negative logFC and white showing no change. Each GO Term is assigned a color corresponding to the color of the ribbon indicating the relationship with the genes.

Functional characterization of the DEG between the urea and control treatments was done with GO analysis using the DAVID software for biological processes, cellular components, and molecular functions (Figure 3).

**Figure 4:**
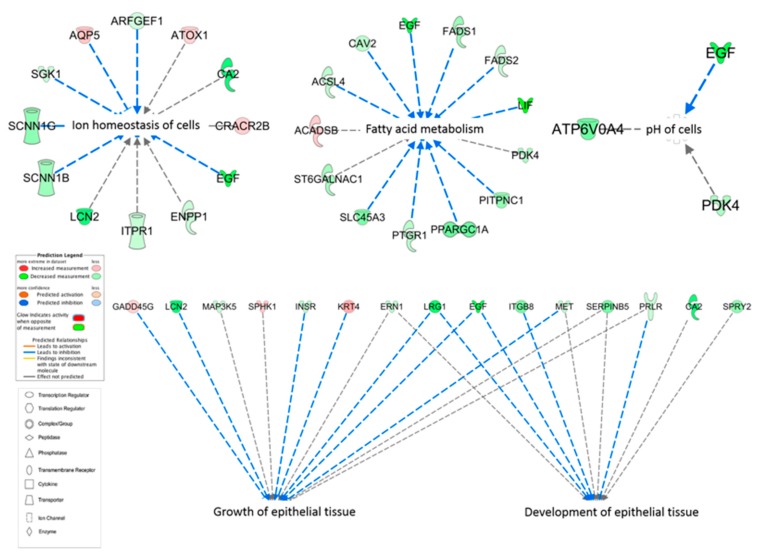
Ingenuity pathway analysis (IPA) of diseases and biological functions of the differentially expressed genes displayed as nodes (genes) and edges (biological relationship between nodes). The color intensity of each node represents fold change expression, red (upregulated), and green (downregulated). The edges connecting the genes to the respective functions represent the predicted relationships, blue representing inhibition and grey representing effect not predicted based on the IPA activation z-scores, combination of directional information encoded by the gene expression with information curated from the literature.

The core analysis of the DEG using IPA showed 501 categories of diseases and biological functions. The categories that had biological interest for this study and a *P* < 0.05 were ion homeostasis of cells (12 genes related, *P* = 0.007), fatty acid metabolism (13 genes related, *P* = 0.000), pH of cells (3 genes related, 0.002), growth of epithelial tissue (11 genes related, *P* = 0.002), and development of epithelial tissue (8 genes related, *P* = 0.002) (Figure 4).

### 3.7. Upstream Regulators

ETS variant 4 (*ETV4*), epidermal growth factor (*EGF*), ETS homologous factor (*EHF*), insulin receptor substrate 2 (*IRS2*), and serum/glucocorticoid regulated kinase 1 (*SGK1*) were predicted as upstream regulators and differentially expressed in our dataset. Their respective target molecules in the dataset are shown in Table 1. 

### 3.8. Weighted Correlation Network Analysis (WGCNA)

**Figure 5:**
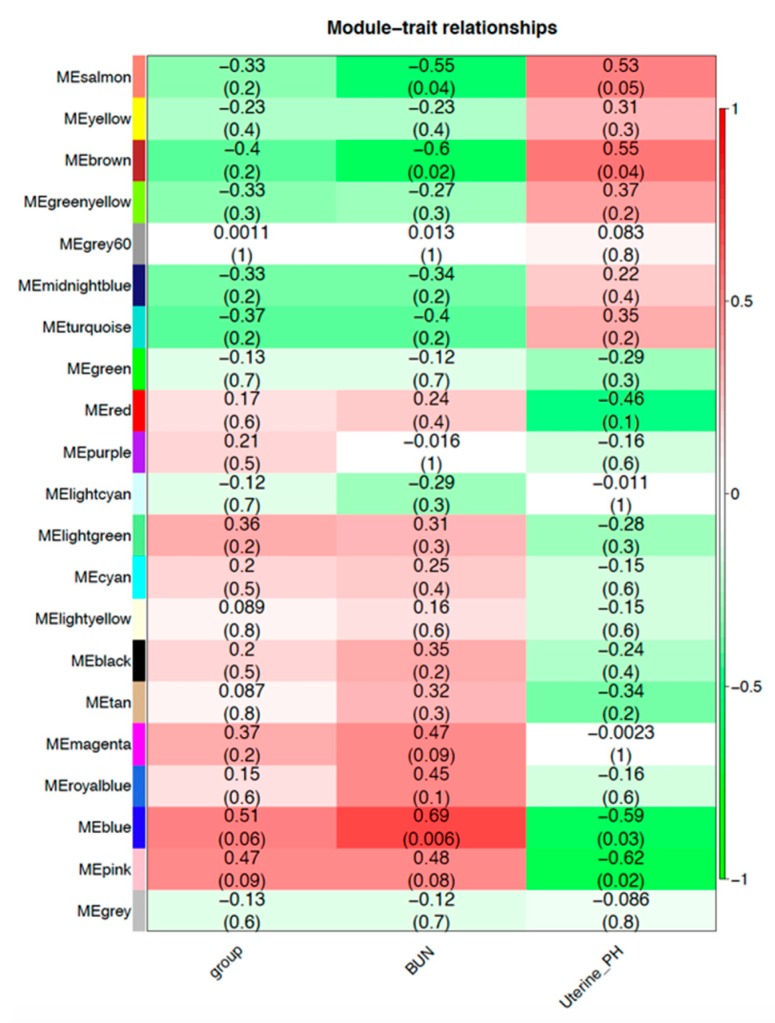
Gene expression modules with weighted correlation network analysis (WGCNA). Matrix with the module-trait relationships and corresponding *p*-values of the modules on the y-axis and selected traits related with treatment on the x-axis. The y-axis is colored according to the correlation, with red representing a strong positive correlation and green representing a strong negative correlation. Traits are: Group (control and urea groups), BUN (blood urea nitrogen concentrations at hour 6 of treatment), and uterine pH (uterine pH measured at hour 6 of treatment).

The WGCNA analysis identified hub genes, pointing out genes that have an important role in the genetic interaction network, by regulating other genes in their module showing that they might be markers for urea-treatment in the endometrium of mares. WGCNA identified 21 modules that were highly correlated to the traits of interest (Figure 5). A list of genes from the modules that had a significant relationship with the traits used is presented in Supplementary Table S3. The Brown Module is of interest, as it had genes with a high membership related to the IPA diseases and biological functions; it had a positive correlation with BUN and a negative correlation to uterine pH. 

### 3.9. Quantitative Real-Time PCR

Analysis of the correlation between genes with RT-qPCR (−ΔCT) and the RNA sequencing results (FPKM) showed that 13 (72.23%) genes had a significant correlation between the two methods. There was a similar pattern of regulation with a significant correlation between −ΔCT and FPKM (Table 2).

## 4. Discussion

To the best of our knowledge, this is the first study to elucidate global changes in mRNA expression profile in the equine endometrium of mares with a high BUN. In this study, diestrus mares received an acute infusion of urea intravenously to elevate BUN and allow evaluation of the endometrial transcriptome. The main findings of the current study were that intravenous infusion of urea resulted in an increase in blood urea nitrogen and a decrease in uterine pH. The functional analyses of changes in the transcriptome in the urea and control groups identified alterations in genes related to pH homeostasis; fatty acid metabolism; and sodium, potassium, and glucose channels. These results serve to illustrate possible effects of a high BUN on the endometrium of mares.

### 4.1. pH of Cells

As expected, the intravenous urea infusion resulted in a significant increase in BUN that was inversely related to uterine pH, similar to studies in cows and ewes [2,5,8,10,11,12]. The IPA disease and functions network associated with the current study indicated that epidermal growth factor (*EGF*), downregulated in the urea group, was related to cell pH. The growth factor *EGF* has an important role in pH changes, inducing an elevation of cytoplasmic pH by modifying the pH sensitivity of the Na+/H+ exchanger [35]. Additionally, the exposure of renal medullary cells to urea stress was similar to *EGF* exposure, corroborating with results relative to urea supplementation changing *EGF* expression [36]. Interestingly, in our dataset *EGF* was identified as an upstream regulator for the DEG and a hub gene for the Brown Module (membership 0.77, *P* = 0.001).

It is worth noting that in vitro studies have shown that a decline in pH of culture media is associated with a decrease in embryo development rate in hamsters [37], mice [38], and cows [15,16]. There was also a lower blastocyst quality in vitro when donor cows received 75 g of urea orally [39]. These changes could explain the decrease in pregnancy rate and embryo development, which have been reported in other species receiving urea or a high protein diet [3,4,40]. 

### 4.2. Solute Carriers and Ion Homeostasis of Cells

Among the DEG, there were five solute carriers: Solute carrier family 25 member 36 (*SLC25A36*), solute carrier family 37 member 1 (*SLC37A1*), solute carrier family 45 member 3 (*SLC45A3*), solute carrier family 52 member 3 (*SLC52A3*), and solute carrier family 6 member 20 (*SLC6A20*). There were also two potassium voltage-gated channels: Potassium voltage-gated channel subfamily A member 3 (*KCNA3*) and potassium voltage-gated channel subfamily C member 4 (*KCNC4*). Solute carriers, such as the ones found in the present study, have been suggested to modify the uterine environment by altering the composition of the uterine fluid and receptivity in implantation [41,42].

Homeostasis of the uterine environment is crucial for uterine functionality; therefore, maintenance of intracellular ionic environment and consequent regulation of fluid is essential and is achieved by water channels, ion channels, and transporters [43,44,45,46]. Previous studies have identified genes that control the uterine fluid environment; for instance, sodium channel epithelial 1 beta subunit (*SCNN1B*) and sodium channel epithelial 1 gamma subunit (*SCNN1G*) encode the epithelial sodium channel, which regulates sodium reabsorption in epithelial cells [47]. Both genes were downregulated in our dataset, similar to a reduction in expression of ENaC channels reported in the endometrium of infertile women [48]. These two genes were also hub genes in the Brown Module, *SCNN1B* (membership = 0.76, *P* = 0.002) and *SCNN1G* (membership = 0.81, *P* = 0.000), suggesting that the urea-treatment might have disrupted the normal ion homeostasis in the endometrium. Additionally, the ion channel ENaC and sodium-potassium ATPases are stimulated by the serum and glucocorticoid regulated kinase 1 (*SGK1*), which regulates ion balance and extracellular fluid volume [49]. A lower expression of *SGK1*, as seen in our study, was reported to alter the local fluid environment leading to reproductive failure in women, possible by a dysregulation in uterine fluid and ion imbalance leading to failure in embryonic implantation [50]. Interestingly, *SGK1* is also an upstream regulator in our dataset. 

Water channels also maintain ion homeostasis, such as aquaporin channels which are permeable to water and ions, regulating reabsorption of endometrial glandular fluid to maintain the luminal fluid volume [44]. The expression of aquaporin 5 (*AQP5*) is progesterone-dependent, showing higher concentrations during high endogenous progesterone stages of the estrous cycle in rats and mares. Additionally, *AQP5* had a high expression in cyclic mares at D8 of diestrus and was upregulated in the endometrium during the time of implantation in rats [51,52,53]. In our dataset, *AQP5* was downregulated in the urea group and was a hub gene in the Brown Module (membership = −0.88, *P* = 0.000). Altogether, we suggest that the urea infusions disrupt the normal function of this channel, resulting in an alteration of the composition and volume of uterine luminal fluid. 

Enzymes are another important mediator of ion homeostasis, as carbonic anhydrase 2 (*CA2*) which was downregulated and also a hub gene in the Turquoise Module (membership= 0.95, *P* = 0.000) in our dataset. *CA2* catalyzes the reversible conversion of carbon dioxide and water to bicarbonate, regulating the acid-base balance and transporting carbon dioxide [54]. The downregulation of *CA2* by the urea-treatment is one of the mechanisms that resulted in an imbalanced uterine environment, supported also by the role that *CA2* has on endometrial gland development in mice and sheep [55].

As shown in the preceding paragraphs, ion channels are crucial for ion homeostasis and one of their regulators are hormones, including progesterone and estradiol [45]. Aldo-keto reductase family 1 member C23 (*AKR1C23*), which is only expressed in the control animals, has a well-known activity of converting progesterone to 20α-hydroxy-4-pregnen-3-one (20α-DHP) and 3α-dihydroprogesterone (3α-DHP) [56,57,58,59]. Therefore, we postulate that the urea-treatment might disrupt the physiological progesterone metabolism that occurs in the endometrium. Consequently, progesterone is not converted into 20α-DHP which in turn affects the hormonal regulation of ion channels in the endometrium and disrupts the normal ion and fluid balance in the uterus of mares. Further studies need to be done to verify this effect of the progesterone metabolism in the endometrium of nonpregnant mares.

### 4.3. Growth and Development of Epithelial Tissue

The endometrium goes through morphological changes during the estrous cycle and early pregnancy [9,60]. The current urea-treatment was responsible for downregulating genes related to the growth and development of epithelial tissue: *EGF*, Serpin B5 (*SERPINB5*), dickkopf WNT signaling pathway inhibitor 1 (*DKK1*), MET proto-oncogene, receptor tyrosine kinase (*MET*), insulin receptor (*INSR*), leucine rich alpha-2-glycoprotein 1 (*LRG1*), prolactin receptor (*PRLR*). The treatment also upregulated angiopoietin like 4 (*ANGPTL4*), keratin 4 (*KRT4*), and growth arrest and DNA damage inducible gamma (*GADD45G*). For example, *EGF* mediates endometrial proliferation [61], while *DKK1* is responsible for initiating endometrial cellular proliferation and differentiation [62]. Additionally, *SERPINB5*, also known as Maspin, plays an important role in embryonic implantation and had a higher expression in the endometrium of pregnant mice when compared to nonpregnant mice [63]. Of interest, *ANGPTL4*, a gene related to angiogenesis and rearrangement of blood vessels [64], had a higher mRNA expression in the endometrial tissue of multiparous pregnant sows when compared to nulliparous animals at day 15 and 25 of pregnancy [65,66]. In mares, there was an upregulation of *ANGPTL4* in the endometrium of day 12 pregnant animals. In the same study, there was a downregulation in *KRT4* in day 12 pregnant mares compared to age-matched nonpregnant mares [66], opposite of what we saw in our dataset with mares during diestrus. Overall, the urea-treatment resulted in a change in expression of genes related to normal endometrial changes, which might result in a disruption of the physiological growth and development of endometrial tissue at this stage of diestrus. However, this hypothesis needs to be tested with further studies.

### 4.4. Fatty Acid Metabolism

An interesting finding was a change in fatty acid metabolism, as was predicted by the disease and function analysis. Fatty acids function as precursors for steroid and eicosanoid synthesis, as well as associated with phospholipids in cell membranes being able to affect uterine function and disturb pregnancy rates [67,68]. Mainly, a significant downregulation of *EGF*, caveolin 2 (*CAV2*), acyl-CoA synthetase long chain family member 4 (*ACSL4*), *SLC45A3*, prostaglandin reductase 1 (*PTGR1*), fatty acid desaturase 1 (*FADS1*), fatty acid desaturase 2 (*FADS2*), and leukaemia inhibitory factor (*LIF*) in the urea group reflected this after the current treatment. 

As eicosanoids are synthetized by fatty acids, the downregulation of the gene *PTGR1,* responsible for encoding catabolic enzymes that degrade eicosanoids, such as prostaglandins, might have a negative effect on the endometrium of animals [69], as an upregulation of it might be paramount for pregnancy, suggested by the upregulation of *PTGR1* in the endometrium of pregnant sows [43] and pregnant mares [66]. Also related to reproduction hormone production in the endometrium, *SLC45A3* enhances long-chain fatty acids and neutral lipid accumulation with a consequent incorporation into cholesterol esters and phospholipids used for steroidogenesis and energy production [70]. 

Furthermore, working through a fatty acid metabolism route, the cytokine *LIF* inhibits lipoprotein lipase activity in vitro [71] which might be related to the fact that *LIF* knockout mice were infertile suggesting that *LIF* codes a protein essential for blastocysts implantation [72]. Additionally, *LIF* was a hub gene in the Brown Module (membership = 0.94, *P* = 0.000) in our dataset.

As previously mentioned, fatty acids are associated with phospholipids of cell membranes [67]. This is related to the *CAV2* gene which encodes a protein that is present in the membrane of caveolin, invaginations of the plasma membrane, responsible for transport of glycolipids [73] and cholesterol across endothelial cells [74]. The resulting downregulation of *CAV2* after the urea-treatment might be due to a deficit in the transport of macromolecules in the endometrium, disrupting the normal fatty acid metabolism in the tissue. Although this work sheds light into an important role of urea-treatment in fatty acids, a clear mechanism of how urea-treatment can affect fatty acid metabolism in the endometrium has not been established and remains to be elucidated. 

## 5. Conclusions

**Figure 6:**
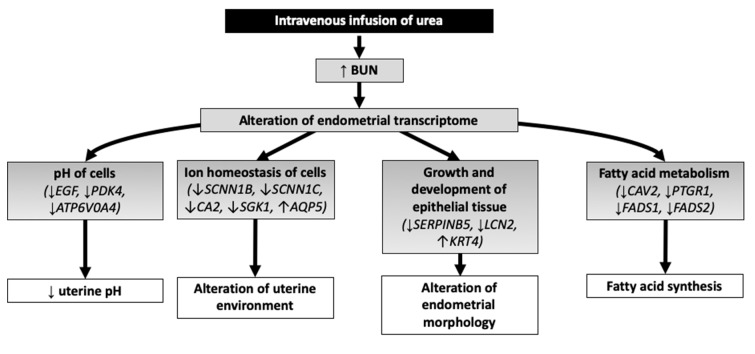
Schematic representation of proposed mechanisms of action of intravenous infusion of urea at day 7 of estrous cycle on the endometrium of mares over 6 h.

Our findings suggest that mares with a high BUN exhibit a decreased uterine pH and changes in gene expression in endometrial tissue are associated with pH regulation, ion channels, changes in the epithelial tissue, and fatty acid metabolism. Specifically, *EGF* appears to play a central role by driving the effects of urea on the endometrial transcriptome (Figure 6). Although this study did not address the effects of elevated BUN on fertility in mares, the changes in gene expression described herein reveal alterations in endometrial function that could be associated with adverse effects on fertility. Therefore, future studies evaluating the effects of elevated BUN in mares during early pregnancy are needed.

## Figures and Tables

**Table 1:** Upstream regulators from the Ingenuity Pathway Analysis (IPA) when comparing the urea and control groups.

Upstream Regulator	Expr Log Ratio	Molecule Type	*p*-Value of Overlap	Target Molecules in Dataset
ETV4	−5.332	transcription regulator	0.024	ETV4, MET
EGF	−3.834	growth factor	0.000	ACSL4, IDS, IGFBP3, LCN2, MAP3K5, PDK4, SLC37A1, SP4, SPHK1, SPRY2
EHF	−1.688	transcription regulator	0.022	ANGPTL4, EHF, MET
IRS2	−1.253	enzyme	0.028	PPARGC1A, THRSP
SGK1	−0.939	kinase	0.000	KCNA3, LIF, SCNN1B, SCNN1G

**Table 2:** Pearson’s correlation of RNA sequencing (Fragments Per Kilobase of transcript per Million mapped reads) and quantitative real-time PCR (−ΔCT) to confirm RNA sequencing results.

Gene	Correlation (R)	*P*-Value	Corrected *P*-Value
ANGPTL4	0.538	0.047	0.846
AQP5	0.840	0.000	0.000
CA2	0.538	0.046	0.828
EGF	0.611	0.020	0.360
ENPP1	0.746	0.002	0.036
ERRFI1	0.836	0.000	0.000
ETV1	0.382	0.177	1.000
FADS1	0.710	0.004	0.072
IGFBP3	0.723	0.003	0.054
INSR	0.419	0.136	1.000
ITGB8	0.677	0.008	0.144
KCNA3	0.393	0.165	1.000
LAMC2	0.402	0.154	1.000
PIGR	0.601	0.023	0.414
PRLR	0.864	0.000	0.000
SERPINA14	0.688	0.007	0.126
SGK1	0.174	0.551	1.000
SPINK7	0.858	0.000	0.000

## Supplementary Materials

The following are available online at https://www.mdpi.com/2073-4425/10/8/576/s1. Table S1: Forward and reverse primers used for quantitative Real-Time PCR analysis. Table S2: List of the most up- and down-differently expressed genes in the endometrium of mares from the urea compared to the control group in a crossover design. Table S3: List of genes identified in modules with a significant relationship with the traits analyzed.
