# Supplementary material for: A High Protein Model Alters the Endometrial Transcriptome of Mares
Source: Genes (Basel). 2019 Jul 30;10(8):576. doi: 10.3390/genes10080576 (PMC6723232; doi:10.3390/genes10080576)
Supplement: Supplementary file 1 [file genes-10-00576-s001.zip › Supplementary files/Supplementary table 2.pdf]

Supplementary table 2. List of the most up- and down-differently expressed genes in the endometrium of mares from the urea compared to the control group in a crossover design.

Gene name	Gene symbol	Orthologous gene (Species)	Log2(fold_change)	P-value	Adjusted p-value
arylacetamide deacetylase	AADAC		-2.476	0.000	0.010
abhydrolase domain containing 15	ABHD15		-0.966	0.001	0.056
abhydrolase domain containing 17C	ABHD17C		-1.424	0.000	0.031
actin binding LIM protein family member 3	ABLIM3		-1.995	0.000	0.031
acyl-CoA dehydrogenase short/branched chain	ACADSB		0.978	0.001	0.064
acyl-CoA synthetase long chain family member 4	ACSL4		-1.102	0.000	0.018
actin, gamma 2, smooth muscle, enteric	ACTG2		3.964	0.000	0.010
ADAMTS like 2	ADAMTSL2		-2.262	0.001	0.100
alpha-fetoprotein	AFP		INFINITE	0.001	0.067
1-acylglycerol-3-phosphate O-acyltransferase 5	AGPAT5		-0.873	0.001	0.086
aldo-keto reductase family 1 member C23	AKR1C23		INFINITE	0.000	0.010
aldolase, fructose-bisphosphate B	ALDOB		-4.479	0.000	0.010
angiopoietin like 4	ANGPTL4		-1.510	0.000	0.036
aquaporin 5	AQP5		1.075	0.000	0.018
ADP ribosylation factor guanine nucleotide exchange factor 1	ARFGEF1		-0.758	0.001	0.089
Rho GTPase activating protein 5	ARHGAP5		-0.837	0.001	0.067
Cdc42 guanine nucleotide exchange factor 9	ARHGEF9		-0.963	0.001	0.073
AT-rich interaction domain 5B	ARID5B		-1.062	0.001	0.070
antioxidant 1 copper chaperone	ATOX1		0.872	0.001	0.094
ATPase 13A3	ATP13A3		-0.923	0.000	0.018
ATPase H+ transporting V0 subunit a4	ATP6V0A4		-2.171	0.000	0.018
BMP and activin membrane bound inhibitor	BAMBI		-1.972	0.000	0.010
BRCA1 associated RING domain 1	BARD1		-1.103	0.001	0.100
B double prime 1, subunit of RNA polymerase III transcription initiation factor IIIB	BDP1		-0.775	0.001	0.086
baculoviral IAP repeat containing 3	BIRC3		-0.959	0.001	0.056
BRCA1 interacting protein C-terminal helicase 1	BRIP1		-2.145	0.000	0.010
complement C3-like	C3		-1.274	0.000	0.049
complement component 4 binding protein alpha	C4BPA		-1.833	0.000	0.041
carbonic anhydrase 2	CA2		-2.977	0.000	0.010
Cdk5 and Abl enzyme substrate 1	CABLES1		-2.137	0.000	0.010
calcium/calmodulin dependent protein kinase II beta	CAMK2B	*Homo sapiens*	-2.487	0.000	0.010
caveolin 2	CAV2		-1.125	0.000	0.018
cadherin 16	CDH16		-1.610	0.001	0.060

cilia and flagella associated protein 74	CFAP74	-1.041	0.000	0.052
C-type lectin domain containing 11A	CLEC11A	1.067	0.001	0.067
C-type lectin domain containing 20A	CLEC20A	-3.145	0.000	0.010
cytokine dependent hematopoietic cell linker	CLNK	-4.270	0.000	0.010
CKLF like MARVEL transmembrane domain containing 4	CMTM4	-1.000	0.001	0.094
contactin 1	CNTN1	-2.026	0.000	0.024
cordon-bleu WH2 repeat protein	COBL	-1.157	0.000	0.052
cytoplasmic polyadenylation element binding protein 4	CPEB4	-1.056	0.000	0.010
calcium release activated channel regulator 2B	CRACR2B	0.927	0.001	0.067
CREB3 regulatory factor	CREBRF	-0.814	0.001	0.087
cysteine rich transmembrane BMP regulator 1	CRIM1	-1.143	0.000	0.024
colony stimulating factor 3 receptor	CSF3R	-1.768	0.001	0.098
cytochrome b-245 alpha chain	CYBA	0.892	0.001	0.087
doublecortin domain containing 2	DCDC2	-1.078	0.000	0.010
integrator complex subunit 6-like	DDX26B	-0.921	0.001	0.056
DENN domain containing 4A	DENND4A	-2.420	0.000	0.036
dickkopf WNT signaling pathway inhibitor 1	DKK1	-0.941	0.001	0.087
dihydropyrimidine dehydrogenase	DPYD	-0.975	0.001	0.060
dual specificity phosphatase 9	DUSP9	-3.352	0.000	0.010
EF-hand domain containing 2	EFHC2	-2.092	0.000	0.018
epidermal growth factor	EGF	-3.834	0.000	0.010
ETS homologous factor	EHF	-1.688	0.000	0.010
elongation factor for RNA polymerase II 2	ELL2	-1.000	0.000	0.044
endonuclease domain containing 1	ENDOD1	-1.969	0.000	0.010
ectonucleotide pyrophosphatase/phosphodiesterase 1	ENPP1	-0.913	0.000	0.031
ectonucleotide pyrophosphatase/phosphodiesterase 4	ENPP4	-0.848	0.000	0.052
EPM2A interacting protein 1	EPM2AIP1	-1.276	0.000	0.010
endoplasmic reticulum to nucleus signaling 1	ERN1	-1.157	0.000	0.018
ERBB receptor feedback inhibitor 1	ERRFI1	-0.871	0.000	0.041
ETS variant 1	ETV1	-2.041	0.000	0.044
ETS variant 4	ETV4	-5.332	0.000	0.010
ETS variant 5	ETV5	-1.441	0.000	0.010
fatty acid desaturase 1	FADS1	-1.602	0.000	0.036
fatty acid desaturase 2	FADS2	-0.998	0.001	0.056
family with sequence similarity 81 member A	FAM81A	-2.252	0.000	0.010
FCH domain only 2	FCHO2	-0.877	0.000	0.044
free fatty acid receptor 4	FFAR4	-3.122	0.000	0.041
focadhesin	FOCAD	1.035	0.001	0.087

growth arrest and DNA damage inducible gamma	GADD45G		0.827	0.001	0.073
polypeptide N-acetylgalactosaminyltransferase 15	GALNT15		-2.339	0.000	0.010
polypeptide N-acetylgalactosaminyltransferase 4	GALNT4		-1.353	0.001	0.084
GLIS family zinc finger 3	GLIS3		-1.070	0.001	0.060
geminin coiled-coil domain containing	GMNC		-1.401	0.000	0.018
granulysin	GNLY		-1.136	0.000	0.031
G protein-coupled receptor 152	GPR152		-1.554	0.000	0.010
G protein-coupled receptor 176	GPR176		-2.491	0.000	0.010
homeodomain interacting protein kinase 3	HIPK3		-0.913	0.001	0.084
heparan sulfate-glucosamine 3-sulfotransferase 1	HS3ST1		-1.317	0.000	0.044
heparan sulfate 3-O-sulfotransferase-4	HS3ST4	*Homo sapiens*	-0.952	0.000	0.052
iduronate 2-sulfatase	IDS		-0.816	0.001	0.086
insulin like growth factor binding protein 3	IGFBP3		-1.206	0.000	0.010
insulin receptor	INSR		-1.154	0.000	0.010
insulin receptor substrate 2	IRS2		-1.253	0.000	0.010
integrin subunit beta 8	ITGB8		-2.041	0.000	0.010
inositol 1,4,5-trisphosphate receptor type 1	ITPR1		-0.974	0.000	0.044
joining chain of multimeric IgA and IgM	JCHAIN		-0.945	0.001	0.084
KN motif and ankyrin repeat domains 4	KANK4		-1.838	0.001	0.070
potassium voltage-gated channel subfamily A member 3	KCNA3		-1.854	0.001	0.080
potassium voltage-gated channel subfamily C member 4	KCNC4		-2.445	0.000	0.010
KIAA1217	KIAA1217		-0.956	0.001	0.094
kinesin family member 12	KIF12		-2.167	0.000	0.010
kinesin family member 5C	KIF5C		-1.011	0.000	0.036
keratin 4	KRT4		1.564	0.000	0.010
keratin 78	KRT78		1.066	0.000	0.010
laminin subunit beta 3	LAMB3		-1.178	0.000	0.036
laminin subunit gamma 2	LAMC2		-1.496	0.000	0.024
lipocalin 2	LCN2		-2.985	0.000	0.049
LIF, interleukin 6 family cytokine	LIF		-4.828	0.000	0.010
MHC class I antigen pseudogene	LOC100054536		0.964	0.000	0.024
xanthine dehydrogenase/oxidase	LOC100054688		-0.980	0.000	0.041
proline rich 4 (lacrimal)- PRR4	LOC100066131	*Equus przewalskii*	-1.684	0.000	0.010
gasdermin-C	LOC100068406		-2.597	0.000	0.010
homeobox protein MSX-3-like	LOC100146619		0.973	0.000	0.041
metallothionein-1A-like	LOC100630794		0.914	0.001	0.073
T-lymphocyte surface antigen Ly-9	LOC102147390		-0.850	0.000	0.041

short/branched chain specific acyl-CoA dehydrogenase, mitochondrial pseudogene	LOC102149005		0.998	0.001	0.067
leucine rich alpha-2-glycoprotein 1	LRG1		-3.278	0.000	0.010
leucine rich repeat containing 26	LRRC26		1.467	0.000	0.010
mannosidase alpha class 1C member 1	MAN1C1		-1.631	0.000	0.010
mannosidase alpha class 2A member 1	MAN2A1		-1.206	0.000	0.024
mitogen-activated protein kinase kinase kinase 5	MAP3K5		-1.087	0.001	0.070
mediator complex subunit 13 like	MED13L		-0.998	0.000	0.018
MET proto-oncogene, receptor tyrosine kinase	MET		-1.074	0.000	0.010
methyltransferase like 17	METTL17		0.807	0.001	0.070
membrane metalloendopeptidase like 1	MMEL1		1.111	0.001	0.100
N-acetyltransferase 8B	NAT8B		-2.576	0.001	0.056
N-myc downstream regulated 1	NDRG1		-1.082	0.001	0.073
nuclear receptor subfamily 1 group D member 2	NR1D2		-1.048	0.000	0.010
oxidation resistance 1	OXR1		-0.856	0.001	0.064
pantothenate kinase 3	PANK3		-1.333	0.000	0.010
papilin, proteoglycan like sulfated glycoprotein	PAPLN		-1.107	0.000	0.010
progestin and adipoQ receptor family member 5	PAQR5		-3.706	0.000	0.010
pyruvate dehydrogenase kinase 4	PDK4		-1.401	0.000	0.010
PDZ and LIM domain 3	PDLIM3		-2.099	0.000	0.010
paternally expressed 10	PEG10		-1.028	0.001	0.077
progastricsin	PGC	*Canis lupus dingo*	0.942	0.000	0.049
polyhomeotic homolog 3	PHC3		-0.829	0.001	0.089
pleckstrin homology like domain family B member 2	PHLDB2		-2.082	0.000	0.010
polymeric immunoglobulin receptor	PIGR		-1.328	0.000	0.010
phosphatidylinositol transfer protein cytoplasmic 1	PITPNC1		-1.725	0.000	0.044
phospholipase C beta 1	PLCB1		-1.283	0.000	0.010
phosphatidylinositol specific phospholipase C X domain containing 3	PLCXD3		-1.396	0.001	0.086
PPARG coactivator 1 alpha	PPARGC1A		-2.211	0.000	0.010
protein phosphatase 4 regulatory subunit 4	PPP4R4		-2.053	0.000	0.010
phosphatidylinositol-3,4,5-trisphosphate dependent Rac exchange factor 2	PREX2		-1.656	0.000	0.010
prolactin receptor	PRLR		-0.995	0.001	0.080
prostaglandin reductase 1	PTGR1		-1.176	0.000	0.024
RAP1 GTPase activating protein 2	RAP1GAP2		-1.474	0.000	0.024
RAS and EF-hand domain containing	RASEF		-1.939	0.000	0.010
RAS guanyl releasing protein 1	RASGRP1		-1.877	0.000	0.010
retinol binding protein 1	RBP1		1.113	0.000	0.031

ring finger protein 208	RNF208		0.848	0.001	0.092
RAR related orphan receptor A	RORA	*Homo sapiens*	-1.585	0.000	0.010
RP11-401A10	RP11-401A10	*Homo sapiens*	-1.212	0.001	0.100
relaxin family peptide receptor 1	RXFP1		-1.518	0.000	0.010
scinderin	SCIN		-1.674	0.000	0.010
sodium channel epithelial 1 beta subunit	SCNN1B		-1.543	0.000	0.010
sodium channel epithelial 1 gamma subunit	SCNN1G		-1.738	0.000	0.010
short chain dehydrogenase/reductase family 16C member 5	SDR16C5		-1.462	0.001	0.089
short chain dehydrogenase/reductase family 42E, member 1	SDR42E1		-1.054	0.000	0.044
semaphorin 4G	SEMA4G		-1.286	0.001	0.080
serine peptidase inhibitor clade A (alpha-1 antiproteinase, antitrypsin) member 14	SERPINA14		-2.851	0.000	0.010
serpin family B member 5	SERPINB5		-2.064	0.000	0.036
serpin family I member 1	SERPINI1		-4.225	0.000	0.010
serum/glucocorticoid regulated kinase 1	SGK1		-0.939	0.000	0.052
serum/glucocorticoid regulated kinase family member 3	SGK3	*Homo sapiens*	-7.348	0.000	0.010
solute carrier family 25 member 36	SLC25A36		-0.862	0.001	0.067
solute carrier family 37 member 1	SLC37A1		-1.079	0.000	0.018
solute carrier family 45 member 3	SLC45A3		-2.007	0.000	0.010
solute carrier family 52 member 3	SLC52A3		-3.983	0.000	0.018
solute carrier family 6 member 20	SLC6A20		-4.538	0.000	0.010
SLIT and NTRK like family member 4	SLITRK4		-3.646	0.000	0.010
antileukoproteinase	SLPI		-2.955	0.001	0.092
Sp4 transcription factor	SP4		-1.394	0.000	0.018
sphingosine kinase 1	SPHK1		1.107	0.000	0.018
alpha-1-antiproteinase 2-like	Spi2-1		-4.304	0.000	0.010
serine peptidase inhibitor, Kazal type 7 (putative)	SPINK7		2.035	0.000	0.010
SPARC (osteonectin), cwcv and kazal like domains proteoglycan 2	SPOCK2		-0.990	0.001	0.094
spondin 2	SPON2		0.882	0.001	0.089
signal peptide peptidase like 2A	SPPL2A		-1.115	0.000	0.010
sprouty RTK signaling antagonist 2	SPRY2		-1.776	0.000	0.010
sperm specific antigen 2	SSFA2		-0.910	0.001	0.087
ST6 N-acetylgalactosaminide alpha-2,6-sialyltransferase 1	ST6GALNAC1		-1.499	0.001	0.086
STEAP family member 1	STEAP1		-1.573	0.000	0.024
sulfotransferase 1C4	SULT1C4		0.949	0.001	0.087
synaptotagmin like 5	SYTL5	*Equus asinus*	-1.649	0.000	0.052
transcription factor EB	TFEB		0.894	0.001	0.056

thyroid hormone responsive	THRSP		1.018	0.001	0.077
transducin like enhancer of split 6	TLE6		1.309	0.000	0.049
transmembrane protein 154	TMEM154		-1.045	0.000	0.044
transmembrane serine protease 2	TMPRSS2		-3.022	0.000	0.010
transmembrane serine protease 4	TMPRSS4		-3.654	0.000	0.010
transient receptor potential cation channel subfamily M member 5	TRPM5		1.846	0.000	0.044
tetraspanin 7	TSPAN7		-1.317	0.000	0.010
tubulin tyrosine ligase like 6	TTLL6		-1.879	0.000	0.010
ubiquitin specific peptidase 53	USP53		-0.984	0.000	0.052
villin like	VILL		-1.049	0.001	0.089
V-set and transmembrane domain containing 5	VSTM5		-1.264	0.000	0.018
XK related 5	XKR5		-1.819	0.000	0.010
XK-related protein 5-like	XKR5-like	*Equus przewalskii*	-6.294	0.000	0.010
